# The molecular mechanism of the anticancer effect of Artonin E in MDA-MB 231 triple negative breast cancer cells

**DOI:** 10.1371/journal.pone.0182357

**Published:** 2017-08-03

**Authors:** Imaobong Christopher Etti, Rasedee Abdullah, Arifah Kadir, Najihah Mohd Hashim, Swee Keong Yeap, Mustapha Umar Imam, Faiqah Ramli, Ibrahim Malami, Kian Lim Lam, Ubong Etti, Peter Waziri, Marsitoh Rahman

**Affiliations:** 1 Pharmacology and Toxicology, Faculty of Veterinary Medicine, Universiti Putra Malaysia, Selangor, Malaysia; 2 Department of Pharmacology and Toxicology, University of Uyo, Uyo, Nigeria; 3 Department of Veterinary Pathology and Microbiology, Faculty of Veterinary Medicine, University Putra Malaysia, Selangor, Malaysia; 4 Department of Veterinary Preclinical Science, Faculty of Veterinary Medicine, Universiti Putra Malaysia, Selangor, Malaysia; 5 Department of Pharmacy, Faculty of Medicine, University of Malaya, Kuala Lumpur, Malaysia; 6 Laboratory of Vaccine and Immunotherapeutics, Institute of Bioscience, University Putra Malaysia, Selangor, Malaysia; 7 School of Public Health, Zhengzhou University, Zhengzhou city, Henan Province, PR China; 8 Institute of Bioproduct Development, Universiti Technologyi Malaysia, Johor, Malaysia; 9 MAKNA-Cancer Research Laboratory, Institute of Bioscience, Universiti Putra Malaysia, Selangor, Malaysia; 10 Faculty of Medicine and Health Sciences, Universiti Tunku Abdul Rahman, Selangor, Malaysia; 11 Department of Biochemistry, Obafemi Awolowo University, Ile Ife, Nigeria; Columbia University, UNITED STATES

## Abstract

Nature has provided us with a wide spectrum of disease healing phytochemicals like Artonin E, obtained from the root bark of *Artocarpus elasticus*. This molecule had been predicted to be drug-like, possessing unique medicinal properties. Despite strides made in chemotherapy, prognosis of the heterogenous aggressive triple negative breast cancer is still poor. This study was conducted to investigate the mechanism of inhibition of Artonin E, a prenylated flavonoid on MDA-MB 231 triple negative breast cancer cell, with a view of mitigating the hallmarks displayed by these tumors. The anti-proliferative effect, mode of cell death and the mechanism of apoptosis induction were investigated. Artonin E, was seen to effectively relinquish MDA-MB 231 breast cancer cells of their apoptosis evading capacity, causing a half-maximal growth inhibition at low concentrations (14.3, 13.9 and 9.8 μM) after the tested time points (24, 48 and 72 hours), respectively. The mode of cell death was observed to be apoptosis with defined characteristics. Artonin E was seen to induce the activation of both extrinsic and intrinsic caspases initiators of apoptosis. It also enhanced the release of total reactive oxygen species which polarized the mitochondrial membrane, compounding the release of cytochrome c. Gene expression studies revealed the upregulation of TNF-related apoptosis inducing ligand and proapoptotic genes with down regulation of anti-apoptotic genes and proteins. A G2/M cell cycle arrest was also observed and was attributed to the observed upregulation of p21 independent of the p53 status. Interestingly, livin, a new member of the inhibitors of apoptosis was confirmed to be significantly repressed. In all, Artonin E showed the potential as a promising candidate to combat the aggressive triple negative breast cancer.

## Introduction

Breast cancer is a complex and heterogeneous disease, constituting an enormous burden to the world at large with increasing mortality rates. It is the most frequently diagnosed cancer and unfortunately the leading cause of cancer death among women [1] with an estimated 1.67 million new cases diagnosed in the year 2012 [2]. Epidemiologic studies have outlined both genetic and non-genetic factors that contribute to the development and progression of breast cancer at all stages. Only between 5 to 10% of breast cancers are hereditary. Between 90 to 95% of breast cancer cases are attributed to environmental factors [3], [4] including age (at menarche, menopause and first pregnancy) [5], diet, smoking and alcohol consumption [6], lack of physical activity [7] and the use of oral contraceptives [8].

Notable among breast cancer cases, are the triple negative breast cancers. These cancers are highly aggressive by nature and are characterized by the absence of hormone receptors [9]. Like other cancers, it shares the unique hallmark of apoptosis evasion while on the path to immortality, a contributor to drug responsiveness [10]. There are two major pathways of apoptosis viz: the extrinsic and mitochondrial (intrinsic) pathways [11]. Both pathways are controlled by a number of gene families which finally lead to the phagocytosis of cells by adjacent cells [12], [13]. In spite of the unique attribute of conventional chemotherapies in inducing apoptosis, many of them are constantly facing resistance coupled with their alarming side effects. Even with the strides to lessen the burden of breast cancer, the prognosis of triple breast cancer is still poor [14] [15]. This flurry encouraged the search into natural sources for relatively safe alternatives to ameliorate the clinical consequences of the hormone negative breast cancer.

Currently, natural products and their derivatives represent more than 50% of the widely used anticancer drugs[16][17]. Artonin E, 5-hydroxy-8,8-dimethyl-3-(3-methylbut-2-enyl)-2-(2,4,5-trihydroxyphenyl) pyrano [2,3-h]chromen-4-one, is a prenylated flavonoid isolated from the stem bark of *Artocarpus elasticus* [18]. This compound has been shown to possess several biological activities including arachidonate 5-lipoxigenase inhibition, antibacterial and anticandicidal [19] antimalarial[20][21], anticancer [22][17] and antiestrogenic [23]. Recently, we reported the mitochondrial dysregulation of Artonin E in ovarian cancer [18] as well as its antiestrogenic capacity [23]. However, the antitumor molecular mechanism of Artonin E in triple negative breast cancer, to the best of our knowledge has not yet been reported. This study, utilizes *in vitro* molecular investigations to elucidate the mechanism of Artonin E in the invasive triple negative breast cancer cell line, MDA-MB 231.

## Materials and method

### Preparation of test agents

Artonin E was extracted from the root bark of *Artocarpus elasticus* and characterized as we previously reported [18]. The standard agents, Tamoxifen and Paclitaxel were purchased from Sigma Aldrich, St. Louis, MO, USA. These agents were dissolved in DMSO and diluted with respective medium with highest final DMSO concentration of 0.1% for the *in vitro* cell culture studies.

### Cell viability study

The colorimetric microculture tetrazolium assay (MTT) was used to study the viability of MDAMB 231 cells in accordance to Mosmann (1983) [24]. Briefly, exponentially growing cells were seeded in a 96-well flat bottom tissue culture plate at a density of 0.5 × 10^4^ cells/well. The cells were thereafter treated after 24 hours incubation with different concentration (1.56 to 100 μM) of Artonin E. After the treatment incubation period (24–72 hours), 20 μL of 5 mg/mL of MTT solution was added to each well and the plate was reincubated for 4 hours to facilitate catalysis by mitochondrial dehydrogenases and then solubilized with 100 μL of DMSO. The amount of purple formazan formed was measured colorimetrically at 570 nm. The experiment was done in triplicate. A nonlinear regression analysis was performed and a dose-response curve was fitted using the GraphPad Prism software. The concentration of each agent that evoked a 50% growth inhibition and the 95% confidence interval were determined using the GraphPad Prism software. The dose-response curve was fitted with the percentage viability calculated from the following formula:

$$\%\;of\;cell\;viability=\;\frac{A_{T}}{A_{C}}\times 100$$ (1)

Where A_T_ is the absorbance reading of treated samples and A_C_ the absorbance of control samples treated with 0.1% of DMSO equivalent.

### Cell morphological study

Acridine orange (AO) and Propidium iodide (PI) double staining assay was used to investigate the morphological changes in Artonin E-treated breast cancer cell. A total of 3 × 10^5^ MDA-MB-231 breast cancer cells was seeded in a six-well plate and allowed to adhere overnight before treatment with various concentrations of Artonin E (3, 10 and 30 μM) at various time periods (24 and 48 hours). After trypsinization, the cells were centrifuged at 2000 rpm (Hettich Universal 32 R centrifuge, DJB Labcare Ltd, UK) for 5 minutes and the pellet was washed with ice-cold PBS, re-centrifuged before suspending in 20 μL of PBS. The cells were thereafter stained on ice with 20 μL dye containing 10 μg/mL of AO with 10 μg/mL of PI. Aliquots of 20 μL of the cell suspension were examined under Carl Zeiss Axioskop plus-2 fluorescence microscope. At least 200 cells in each of three fields were immediately assessed for viability, early, late apoptosis and necrosis [12]. The experiment was carried out in triplicate.

### Annexin V-FITC assay

The annexin V-FITC assay was performed using the Annexin V Kit (BD Pharmingen, USA) to investigate apoptosis via detecting externalized phosphatidylserine. Briefly, after trypsinization, the treated cells in the 6-well plate was collected, washed with ice-cold PBS, resuspended in 1X binding buffer before transferring to BD Falcon flow cytometry tubes. Next, 5μL each of PI solution and annexin V-FITC conjugate were added to the cell gently mixed and incubated for 20 minutes at room temperature in the dark. The cells were finally subjected to flowcytometric analysis using laser emitting excitation light at 488 nm and a BD flow cytometer equipped with an Argon laser (Cyan ADP, DAKO, Glostrup, Denmark). The experiment was carried out in triplicate and the data were analyzed using the Summit V4.3 software.

### DNA fragmentation analysis

Qualitative DNA fragmentation (Roche) kit was used in this analysis. Briefly, DNA from Artonin E treated and untreated MDA-MB 231 cells were extracted in accordance with the manufacturer’s protocol. The quality of the extracted DNA was assessed with a nanodrop spectrophotometer (nanodrop lite spectrophotometer, Thermo scientific, USA). The extracted DNA sample was mixed with 1X loading dye and run on 1% agarose gel at 75 V for 1 hour in triplicate. The fragmented DNA was visualized under UV transilluminator and photographed under Gel Doc (Bio-rad, Hercules, CA, US).

### Flowcytometry cell cycle analysis

The cell cycle analysis was investigated using the BD Cyclestest Plus DNA Kit (BD Biosciences, San Jose, CA, US) according to the manufacturer’s instruction. Briefly, the cells was seeded in a T25 tissue culture flask at a density of 1 × 10^6^ cells/flask and allowed to stand overnight in the incubator for attachment prior to treatment with 3, 10 and 30 μM of Artonin E. Treated cell were trypsinized, collected and centrifuged at 3000 rpm (Hettich Universal 32 R centrifuge, DJB Labcare Ltd, UK) for 5 minutes. The supernatant was discarded and the cells were washed twice with 1 mL of buffer consisting of sodium citrate, sucrose and DMSO. For the staining procedure, the cell suspension was transferred into a flow cytometry tube and 250 μL of solution A (trypsin buffer) was added to each tube, mixed gently and allowed to react for 10 minutes at room temperature. Solution B, consisting of trypsin inhibitor and RNase buffer was thereafter added to the same tubes and allowed to incubate for another 10 minutes at room temperature. Two hundred microliters of ice-cold solution C comprising of propidium iodide (PI) was then added to the same tubes mixed by gentle tapping and allowed to incubate in the dark on ice for another 10 minutes. The stained MDA-MB 231 samples were directly analyzed using BD FACS Calibur flow cytometer (Becton Dickson, Biosciences, San Jose, CA, US). The experiment was carried out in triplicate.

### Caspase 8 and 9 fluorimetric assay

The activities of caspases 8 and 9 in the breast cancer cells were determined using a commercial fluorimetric assay kit (R&D System) based on spectrophotometric detection. Briefly, the MDA-MB-231 cells were seeded in T25 flask at a density of 1 × 10^6^ cells/flask. After attachment, the cells were treated with 3, 10 and 30 μM of Artonin E for 24 hours. The cells were trypsinized and centrifuged at 2,500 rpm Hettich Universal 32 R centrifuge, DJB Labcare Ltd, UK) in a conical tube for 10 minutes. The cell pellet was lysed by the addition of 50 μL of cold lysis buffer containing 10 μg/mL of Aproptinin, μg/mL of Leupeptin and 10 μg/mL of Pepstatin. The cell lysates were incubated on ice for 10 minutes and then centrifuged at 13,000 rpm (Eppendorf 5424 microcentrifuge, USA) for 1 minute before protein quantification using Pierce BCA Protein Assay Kit. Two hundred microgram of protein in 50 μl solutions from each of the samples was added to a 96 well flat black bottom microplate, followed by the addition of 50 μL 2X reaction buffer 8 or 9, as appropriate, containing 10 μL dithiothreitol (DTT)/mL reaction buffer. For each reaction well, 5 μL of either caspase 8 or 9 fluorogenic substrate (LEHD-AFC) was added and the plate was incubated at 37°C for two hours. Control wells were without treatment. Three independent experiments were carried out. Finally, the plate was read with a fluorescence microplate reader at an excitation of 400 nm and emission of 505 nm.

### Measurement of reactive oxygen species (ROS) production

To evaluate the production of total ROS, the total ROS assay kit (ebioscience Inc, Affymetrix) was employed in accordance with the manufacturer’s instructions. Briefly, the breast cancer cells at a density of 1 × 10^6^ cells/T25 flask were treated with 3, 10 and 30 μM of Artonin E for 24 hours, trypsinized and centrifuged at 2000 rpm (Hettich Universal 32 R centrifuge, DJB Labcare Ltd, UK) for 5 minutes. The cells were resuspended in PBS and incubated in 100 μL of ROS assay stain in buffer solution in a 37°C incubator for 60 minutes. The cell sample was then analyzed by flow cytometry.

### Multiplex mRNA expression analysis

#### RNA isolation

A 3 mL suspension containing 3 × 10^5^ MDA-MB 231 breast cancer cells were transferred into each well of a 6-well plate and treated for 24 hours with 3, 10 and 30 μM of Artonin E. Total RNA was extracted from the cells using the Total RNA extraction kit (GF-1 TRE kit, Vivantis technologies) according to the manufacturer’s protocol. The purity and concentration of the isolated RNA were ascertained using a spectrophotometer (Beckman Coulter), USA.

#### Reverse transcription and polymerase chain reaction

The reverse transcriptase quantitative PCR (RT-qPCR) was carried out according to the GenomeLab GeXP Start Kit (Beckman Coulter, USA) protocol, in an XP Thermal Cycler (Bioer Technology, Germany). The reaction was set to run for 1 minute at 48°C, 60 minutes at 42°C and 5 minutes at 95°C. The cDNA produced was amplified in a PCR reaction consisting of 2 mL of 5X PCR buffer, 2 μL magnesium chloride, 1 μL of forward primers mixture (Table 1), 0.35 μL of Taq polymerase, and 4.65 μL cDNA. This reaction was run on the XP Thermal Cycler with an initial denaturation at 95°C for 10 minutes, followed by two-step cycles at 94°C for 30 seconds and at 55°C for 30 seconds, and ending with a single-extension cycle at 68°C for 1 minute. The primers for the genes of interest and housekeeping gene (Table 1) were designed on NCBI website and purchased from Biosune (Shanghai, China), while the internal control (Kanr) was supplied by Beckman Coulter (USA).

**Table 1:** The accession number and sequence of the primers used in the gene expression studies.

Genes	Accession Number	Forward primers	Reverse Primers
**Caspase 7**	NM_033338	5' AGGTGACACTATAGAATAGCTTCTTATGTTACCCAGAT 3'	5' GTACGACTCACTATAGGGAGGTGACATTTTTCTTCTTCT 3'
**Caspase 8**	NM_001228	5' AGGTGACACTATAGAATAGTGCGTCCACTTTCTG 3'	5' GTACGACTCACTATAGGGAGGCCAGATCTTCACTGT 3'
**Caspase 9**	NM_032996	5' AGGTGACACTATAGAATAGCTGGTGGAAGAGCTG 3'	5' GTACGACTCACTATAGGGACTCTAAGCAGGAGATGAACA 3'
**Cyclin E**	NM_001238	5' AGGTGACACTATAGAATACAGGATCCAGATGAAGAA 3'	5' GTACGACTCACTATAGGGACCTTAAGTATGTCTTTTCCTT 3'
**P21**	NM_000389	5' AGGTGACACTATAGAATAAGCTGAGGTGTGAGCAG 3'	5' GTACGACTCACTATAGGGACCCAGGCGAAGTCAC 3'
**ACTB**	**NM_001101**	**5' AGGTGACACTATAGAATAGATCATTGCTCCTCCTGAGC 3'**	**5' GTACGACTCACTATAGGGAAAAGCCATGCCAATCTCATC 3'**

#### Separation of PCR products by GeXP genetic analysis system

The PCR products were separated by capillary gel electrophoresis and analyzed using the GeXP machine (Beckman Coulter, Inc.) and the results were analyzed with the fragment analysis module of the GeXP system software and normalized with β-actin on the eXpress Profiler software.

### Proteome profiling of human apoptotic—related proteins

To investigate the effects of Artonin E on the expression pattern of human apoptosis-related proteins, the proteome profiling was investigated using the human apoptosis array kit (R&D Systems, USA), according to the manufacturers’ protocol. The proteins from the treated and untreated breast cancer cells were harvested and quantified using the BCA pierce protein quantification kit. The membranes were first blocked in the Array Buffer 1 in a 4-well multi-dish with 1-hour incubation on a rocking platform shaker. At the same time, 200 μg of protein from each sample was incubated in array buffer 1 at room temperature for 60 minutes. After blocking the membranes, the array buffer 1 was aspirated; the prepared protein samples were added and incubated with the membrane at 4°C overnight on a rocking platform shaker. The following day, each array was carefully removed and washed thrice with 1X washing buffer on a rocking platform. For each of the arrays, 15 μL of the reconstituted biotinylated detection antibody cocktail diluted to 1.5 mL with 1X array buffer 2/3 was used to incubate each array for 1 hour on a rocking platform. Thereafter, the arrays were again washed thrice with 1X array buffer. A 1:2000 dilution of the streptavidin-HRP in 1X Array Buffer 2/3 was used to incubate each of the membrane for 30 minutes on a rocking platform shaker. Following incubation, the arrays were washed thrice with 1X wash buffer. Finally the membranes were carefully removed, placed in a sheet protector and layered with chemiluminiscence reagent before viewing them with the ChemiDoc XRS (Bio-rad, USA). The experiment was done in three replicate.

### Western blot

#### Isolation of total protein

Total protein was extracted from 1 × 10^6^ cells/T25 of MDA-MB-231 cells after 24 hours of treatment with 3, 10 and 30 μM of Artonin E. Briefly, treated and untreated samples were lysed with 100 μL RIPA lysis buffer containing 1 μL of E-64 protease inhibitor cocktail. The pellet was resuspended thoroughly and incubated at 4°C for 30 minutes with agitation before centrifuging it at 10,000 rpm (Eppendorf 5424 Microcentrifuge, USA) for 10 minutes. The supernatant was thereafter transferred into fresh Eppendorf tubes and the concentration of the extracted proteins was quantified using Pierce BCA protein assay kit by microplate assay (universal microplate reader, Biotech, Inc, USA). This was done in three replicates.

#### Protein separation

The protein sample (20μg) was separated by electrophoresis on an SDS-polyacrylamide gel [25] which was subjected to an electrical current of 100 V for 30 minutes, followed by 150 V for 1 h to separate the proteins.

#### Semi-dry transfer and immunodetection

The separated proteins were transfered onto a polyvinylidene difluoride membrane (Bio-rad, Hercules, CA, US) using a semi-dry transblot turbo (Bio-rad, Hercules, CA, US). The electrotransfer was performed for 15 minutes at 15 V per blot of 1.0 mm thick gels. Upon completion of the transfer, the membrane was washed with water for 5 minutes and blocked with 5% nonfat milk in 1X PBST for 1 hour with agitation on a Belly Dancer^®^ (Stovall, Life Science Incorporated, North Carolina, USA). The membrane was thereafter washed thrice in TBST with agitation and blocked overnight at 4°C with the respective specific primary antibody to subunit Bcl-2, Bax, livin, p53 and p21 at 1:1000 dilution in TBST with constant agitation. The target proteins were finally detected after labelling with goat—anti-mouse IgG conjugated to horseradish peroxidase. The immunoreacted protein bands were developed and detected using a chemiluminescence blotting substrate kit (ECL Western blot substrate, Abcam, England). A chemiluminescence image analyzer system (Chemi-Smart, Vilber Lourmat, Germany) was thereafter used to view the membranes and the intensities of band were quantified with the Image Lab software (BioTechniques,USA).

### Statistical analysis

The results are expressed as mean ± SD for at least three replicate analyses for each sample in each assay. Data analysis was performed using GraphPad Prism 5.0 (GraphPad Software Inc., La Jolla, CA, USA). One way analyses of variance was performed, followed by Turkey’s post hoc tests to compare replicate means of treatment and control groups. The significance was set at p<0.05.

## Results

### Artonin E inhibits the proliferation of MDA-MB 231 cancer cells

**Fig 1:**
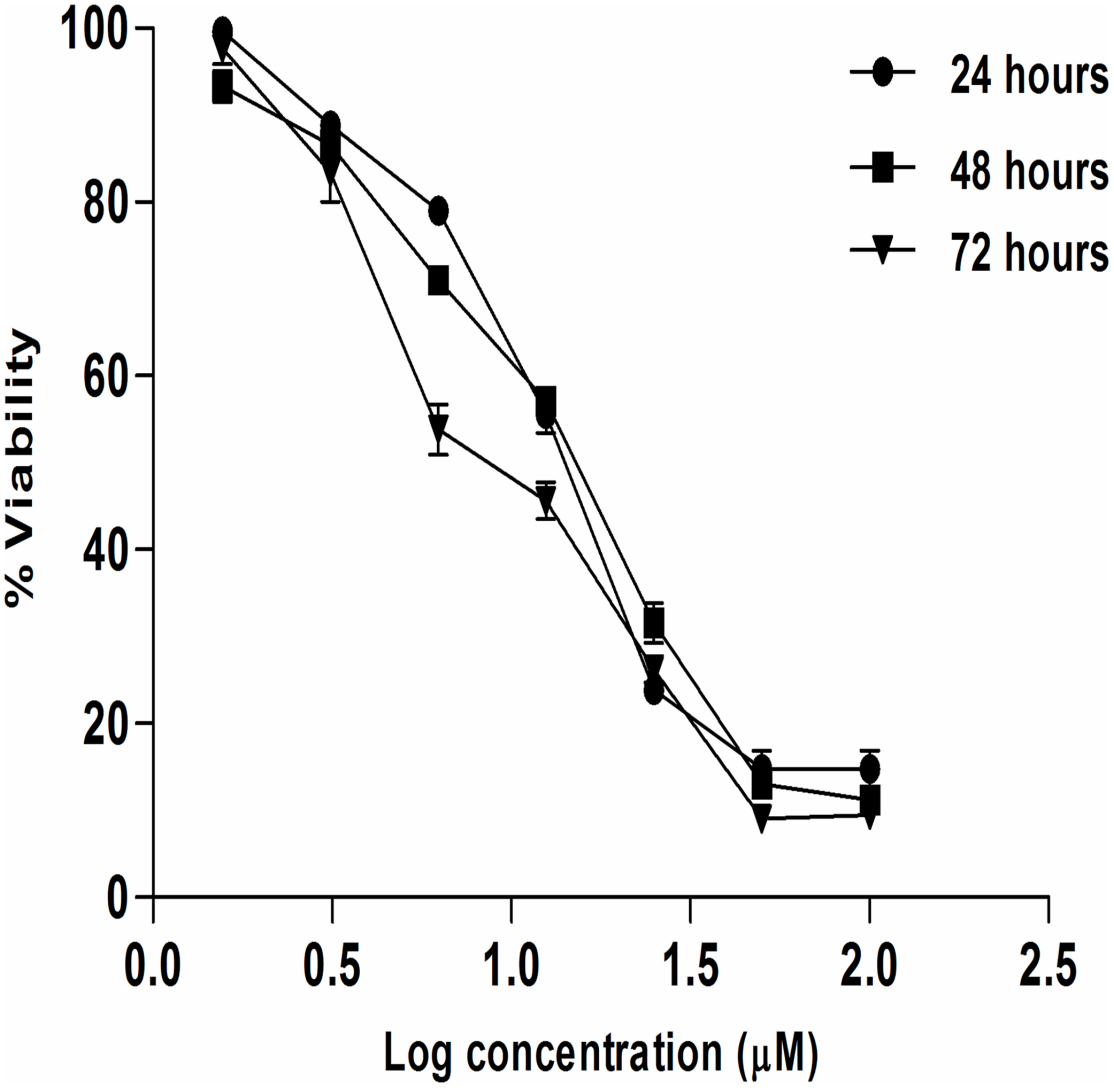
The percentage of MDA-MB-231 cell line viability after treatment with Artonin E. The experiment was done in triplicate at 24, 48, and 72 hours and each point are presented as mean ± SD.

The growth inhibitory effect of Artonin E was investigated on MDA-MB 231 breast cancer cells as well as on the normal breast epithelial cells. From the results, Artonin E, showed a significant (p<0.05) time dependent growth inhibitory effect with half maximal inhibitory concentrations (IC_50_) of 14.13, 13.93 and 9.77 μM at 24, 48 and 72 hours, respectively (Fig 1).

The cytotoxic effect of standard agents, Paclitaxel and Tamoxifen were also determined as well as their 95% confidence interval (Table 2). Artonin E was also observed to be more cytotoxic and selective towards the cancer cells (selectivity index of 4.68) than Tamoxifen with a selectivity index of 1.08.

**Table 2:** The average half-maximal inhibitory concentrations (IC_50_) of Artonin E, and standard agents, Tamoxifen and Paclitaxel on MDA-MB 231 and MCF-10A cell lines.

Cell lines	Artonin E		Paclitaxel		Tamoxifen	
	IC_50_ (μM)	95% CI	IC_50_ (μM)	95% CI	IC_50_ (μM)	95% CI
**MDA-MB 231**	9.77±0.50	8.59–11.12	0.05±0.00	0.03–0.11	23.21±0.86	21.49–25.07
**MCF-10A**	45.80±3.60	42.28–49.60	1.01±0.01	0.73–1.41	25.00±0.30	22.29–28.04

With the exception of those expressed as ranges, all values are presented as means ± standard deviation. IC_50_ = half maximal growth inhibition; CI = confidence interval.

### Artonin E induced apoptosis in MDA-MB 231 breast cancer cells

#### Artonin E induced chromatin condensation and nuclear fragmentation in MDA-MB 231 cancer cells

**Fig 2:**
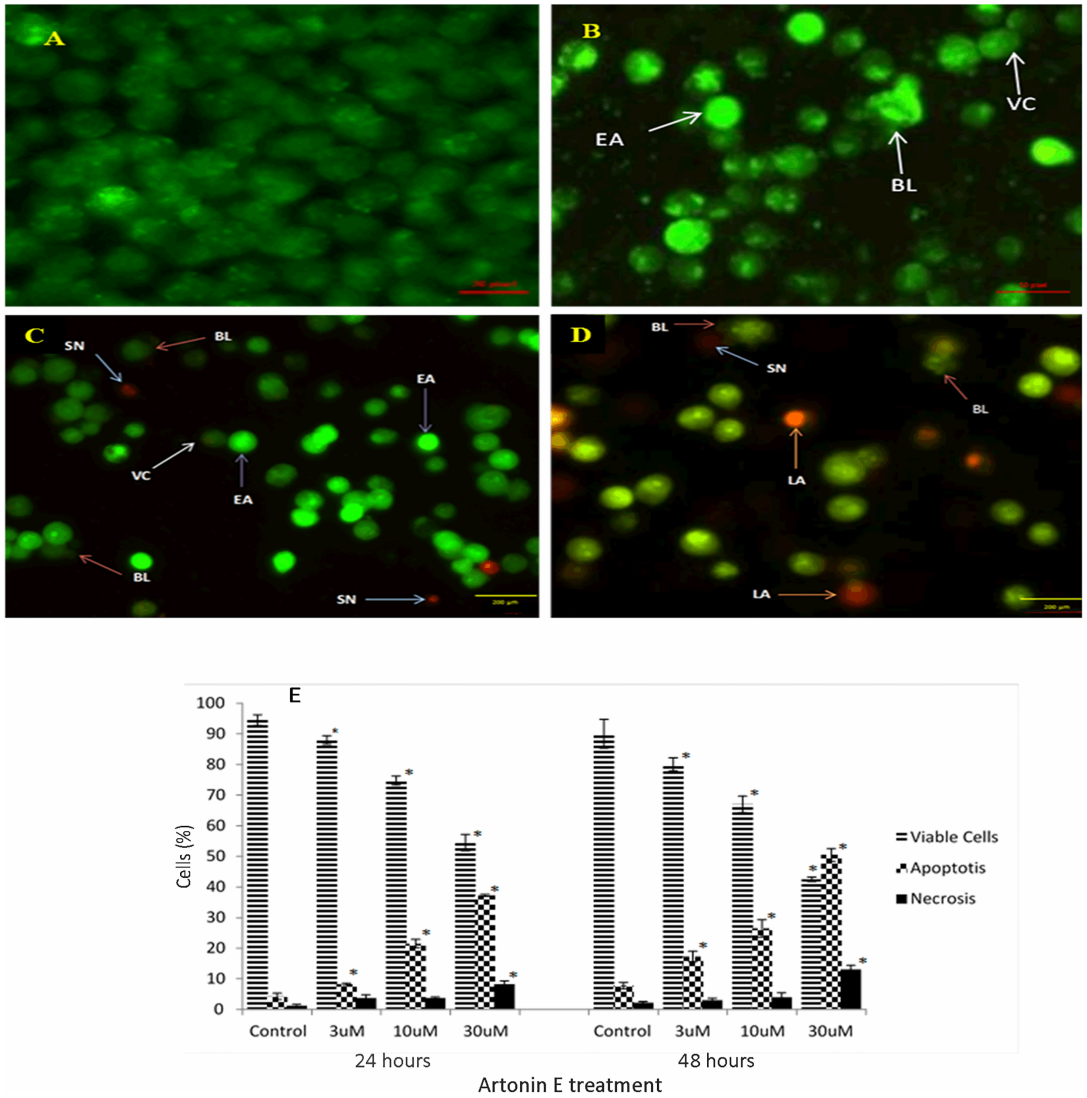
Acridine orange and propidium iodide double staining of MDA-MB 231 breast cancer cells after 24 hour exposure. (A) Control, (B) 3 μM Artonin E. (C) 10 μM Artonin E, (D) 30 μM Artonin E and (E) Quantification of apoptotic morphology at 24 and 48 hours. Each result is presented as mean ± SD of three replicates. * indicates significant difference from the control of each phase (p<0.05). VC = Viable cells; BL = Cell membrane blebbing; CC = chromatin condensation; EA = Early apoptosis; LA = late apoptosis; MN = marginated nuclear chromatin; SN = secondary necrosis. Magnification X200.

The acridine orange (AO) and propidium iodide (PI) double staining analysis revealed the morphology of apoptosis in Artonin E-treated MDA-MB 231 breast cancer cells in a concentration- and time-dependent manner in comparison to the untreated cells. The affected cells exhibited characteristic features of apoptosis like chromatin condensation and membrane blebbing (Fig 2). The normal nuclear structure of the untreated cells was displayed as green fluorescence, whereas bright green fluorescence was shown in early apoptotic cells, caused by interposition of acridine orange with the fragmented DNA. The binding of propidium iodine to denatured DNA was identified by reddish-orange color indicating the late stage of apoptosis. The proportion of viable MDA-MB 231 cells after treatment with Artonin E decreased from 92% in the control to 88% after 24 hours of treatment with 3 μM of Artonin E (Fig 2). However, when the concentration of Artonin E was increased to 10 and 30 μM, the effect was more pronounced, with a significant reduction (p<0.05) in the proportion of viable cells from 92% to 76% and 54% respectively. There was also a corresponding significant (p<0.05) increase in the proportion of cells undergoing apoptosis as the incubation time increased from 24 to 48 hours.

### Externalization of phosphatidylserine and DNA fragmentation following Artonin E treatment

**Fig 3:**
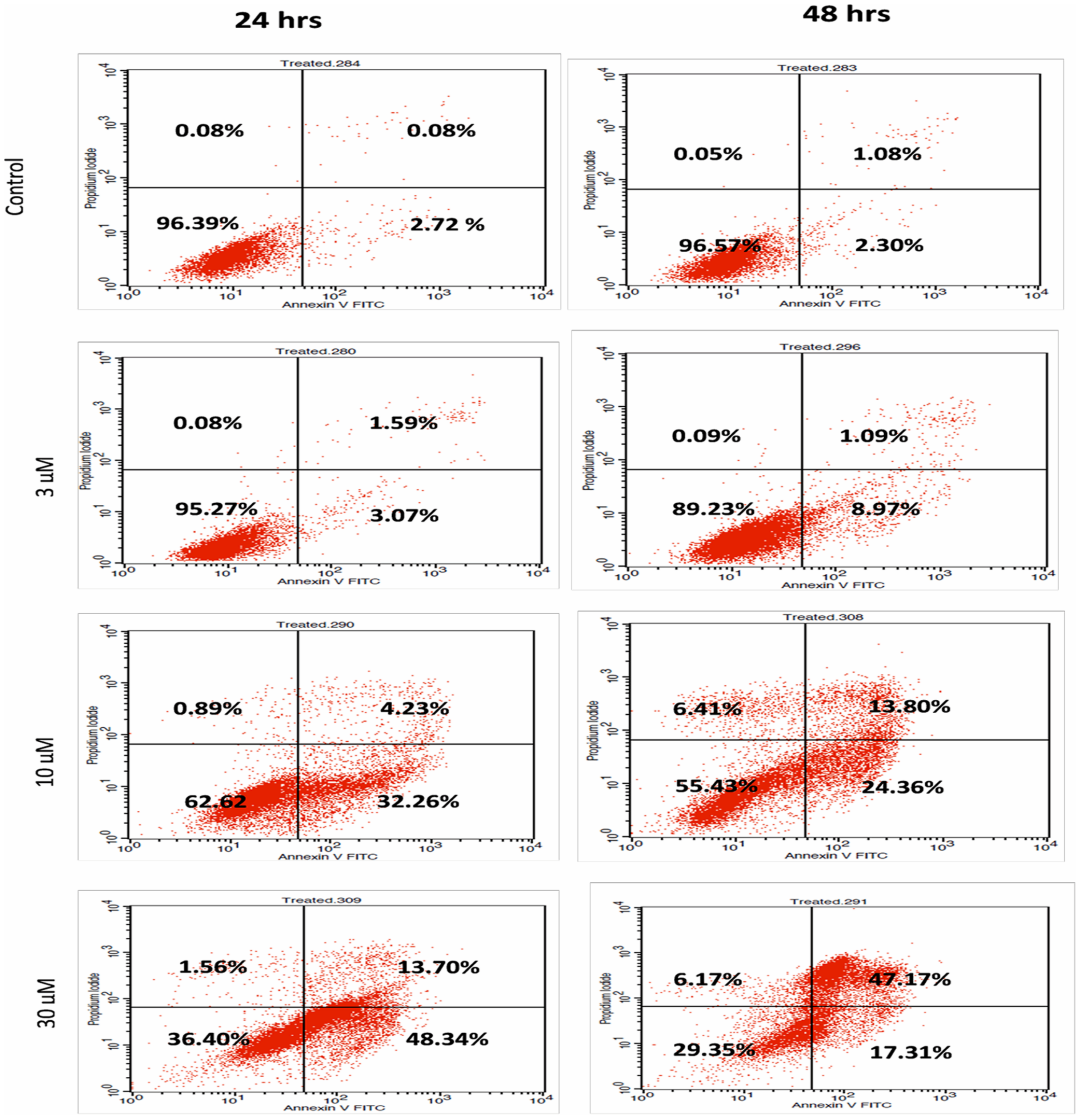
Representative histogram analysis of the annexin V-FITC in flow cytometry assay in MDA-MD-231 cells after 24-hour and 48 hour treatment with Artonin E. Cells population in lower left quadrant are viable, in lower right quadrant are cells at early apoptosis, in upper right quadrant at late stage of apoptosis, and in upper left corner are cells at the necrotic stage.

With the loss of plasma membrane integrity, the phospholipid, phosphatidyl serine (PS) translocates to the outer membrane leaflet and become exposed to the extracellular environment. This exposed phosphatidyl serine was investigated with the annexin V FITC assay, which has high affinity for the phospholipid. From the result, after 24 hours, there was a significant (p<0.05) increase in apoptosis in MDA-MB-231 cells when the concentration of Artonin E was increased to 10 and 30 μM. Artonin E treatment of 3 μM did not cause any significant increase (p>0.05) in early apoptosis when compared with the control. This apoptosis inducing effect was enhanced as the time interval increased to 48 hours, post treatment (Fig 3).

**Fig 4:**
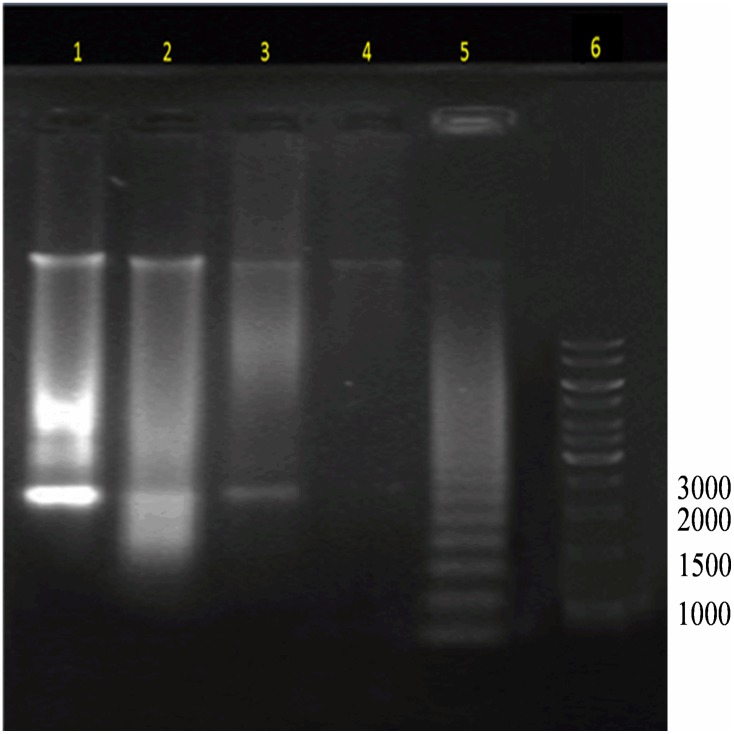
DNA fragmentation of MDA-MB 231 breast cancer cells analysed in 1% agarose gel after 24 hours incubation with different concentration of Artonin E. Lanes 1–3 represents 3, 10 and 30 μM of Artonin E, lane 4 is untreated cancer cells, lane 5 is positive control well treated with 4μg/mL camptothecin and lane 6 is a 1kilobase DNA ladder.

During apoptosis, nucleases are activated to cleave internucleosomal DNA into oligomers of 180 to 200 bp. These fragments can be visualized using agarose gel electrophoresis and verifies the occurrence of apoptosis [26]-[27]. From the results, DNA fragments were visible in all the tested concentrations of Artonin E (Fig 4) and the positive control (lanes 1–3 & 5) while the untreated cells showed no DNA fragmentation (lane 4).

### Artonin E induced G2/M breast cancer cell cycle arrest

**Fig 5:**
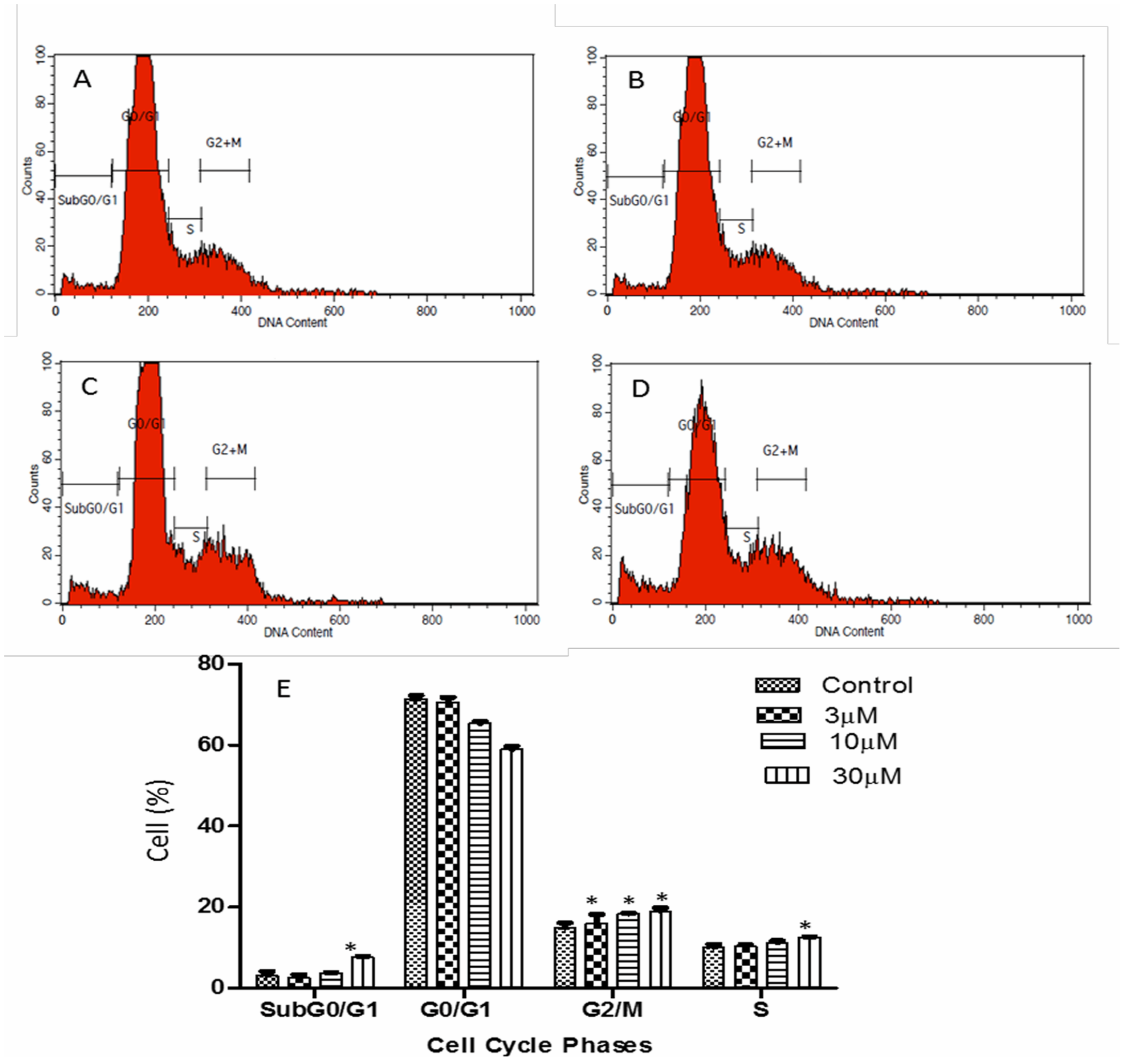
MDA-MB 231 breast cancer cell cycle regulation after 12 hours treatment with (B) 3 μM, (C) 10 μM, and (D) 30 μM Artonin E. (A) is untreated control. (E) Analysis of cell population in the cycle phases. Values are mean ± standard deviation of three replicates. *Means in each cell cycle phase significantly (p<0.05) different from the control.

Deregulation of the cell cycle is a notable hallmark of cancer, however, after 12 hours of incubating MDA-MB 231 breast cancer cells with Artonin E, the percentage of cells in the G2/M phase increased marginally from 14.8% in the untreated control to 16.0, 18.3 and 19.05% after treatment with 3, 10 and 30 μM Artonin E, respectively (Fig 5). There was also a significant (p<0.05) accumulation of cells in the sub G0/G1 phase, indicating the population of cell death.

**Fig 6:**
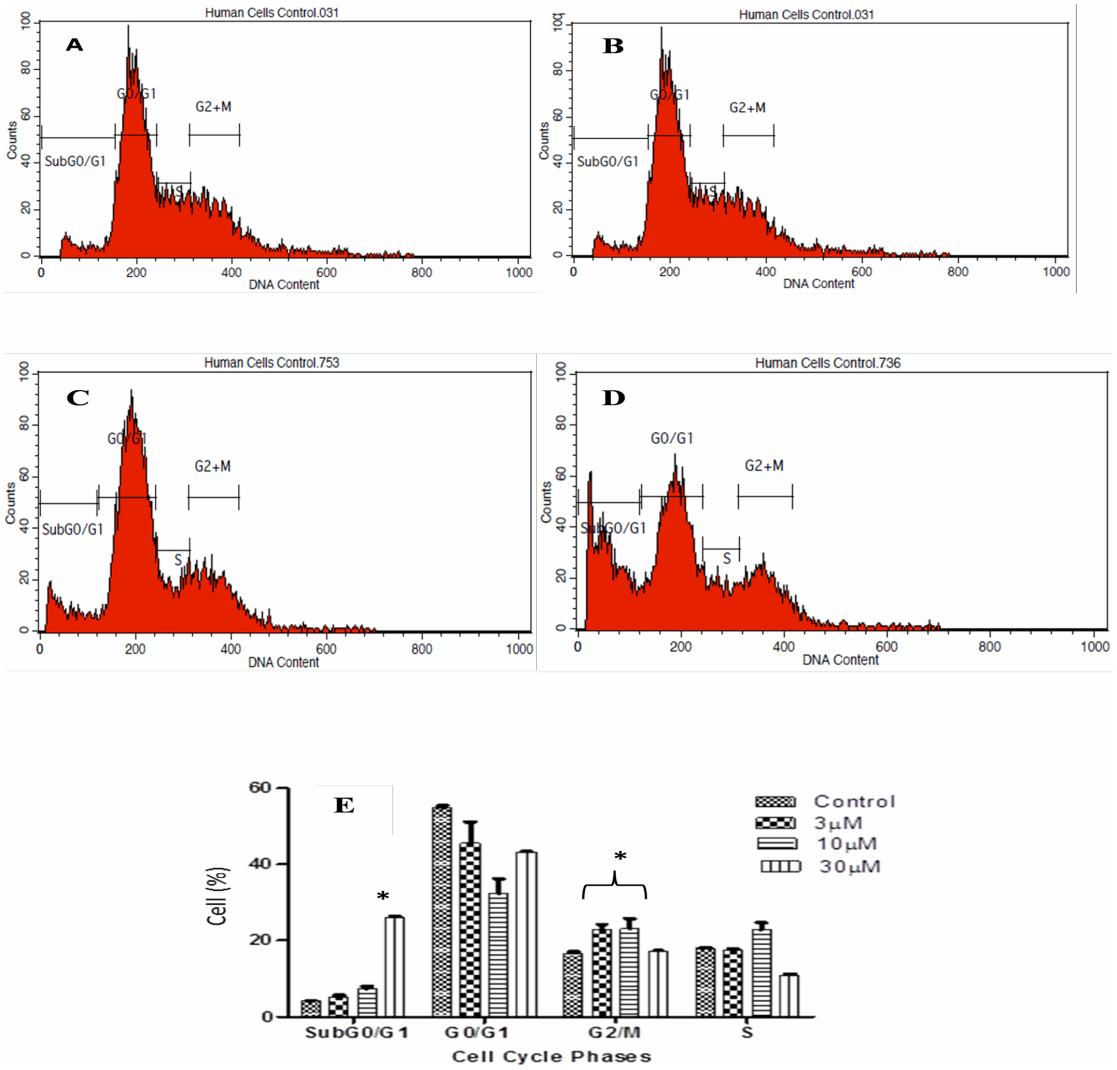
MDA-MB 231 breast cancer cell cycle regulation after 24 hours treatment with (B) 3 μM, (C)10 μM, and (D) 30 μM Artonin E. (A) is untreated control. (E) Analysis of cell population in the cycle phases. Values are mean ± standard deviation of three replicate. *Means in each cell cycle phase significantly (p<0.05) different from the control.

Upon increasing the time of exposure to 24 hours, the cells in the sub G0/G1 population increased significantly with a transient accumulation at the G2/M phase at 3 and 10 μM concentrations of Artonin E (Fig 6).

### Artonin E activates both caspases 8 and 9 and enhances the production of total reactive oxygen species (ROS)

**Fig 7:**
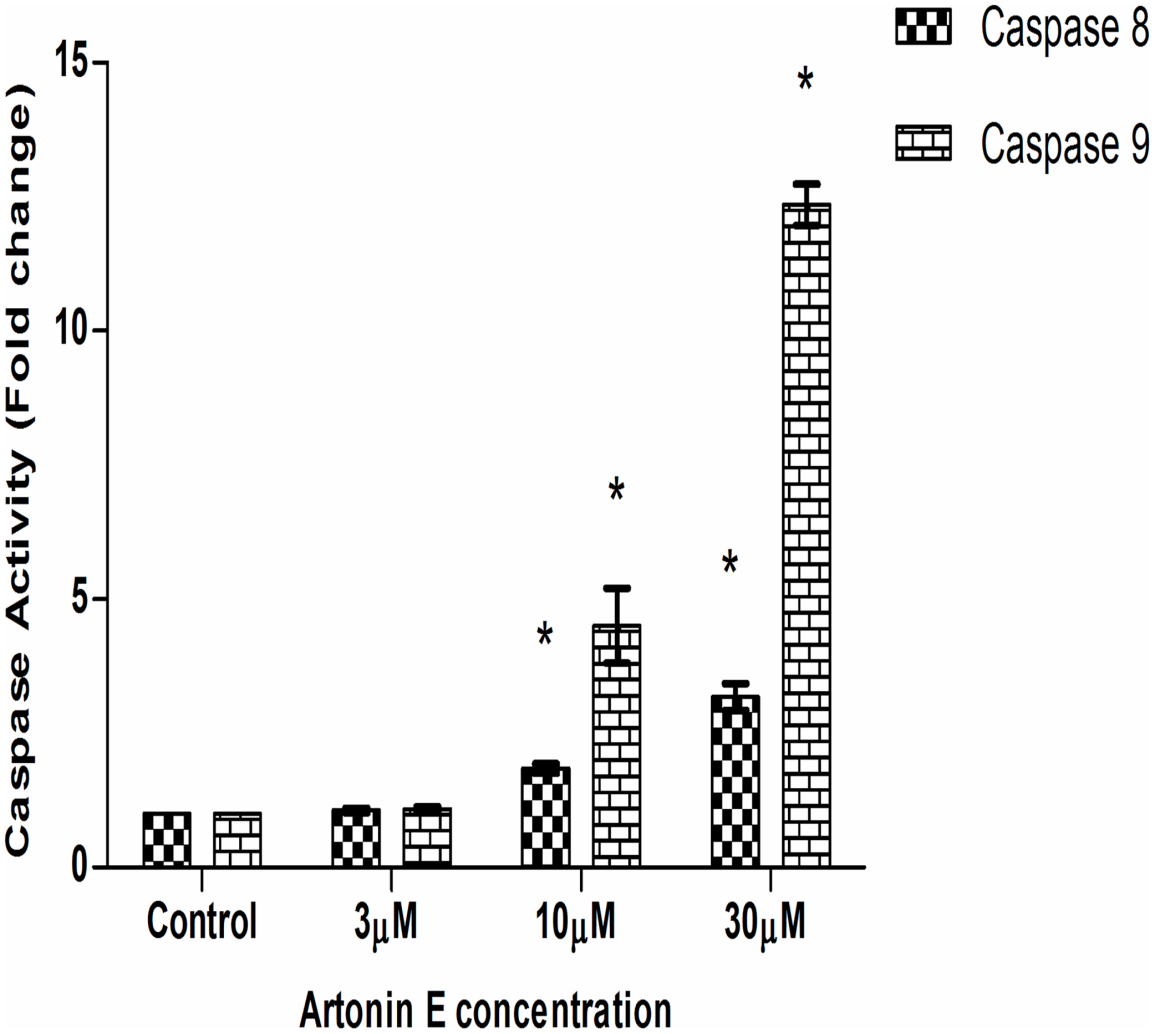
Detection of Caspases 8 and 9 activations in MDA-MB-231 cells treated with Artonin E after 24 hours. Values are presented as mean ± standard deviation of three replicates. *Means differ significantly (p<0.05) from the untreated control.

Caspases are major players of apoptosis. Caspases 8 and 9 are the initiators of extrinsic and intrinsic pathway of apoptosis respectively. From the results, after 24 hours exposure of the MDA-MB 231 cancer cells to Artonin E, there was a dose dependent increase in the activities of both caspases 8 and 9 (Fig 7).

**Fig 8:**
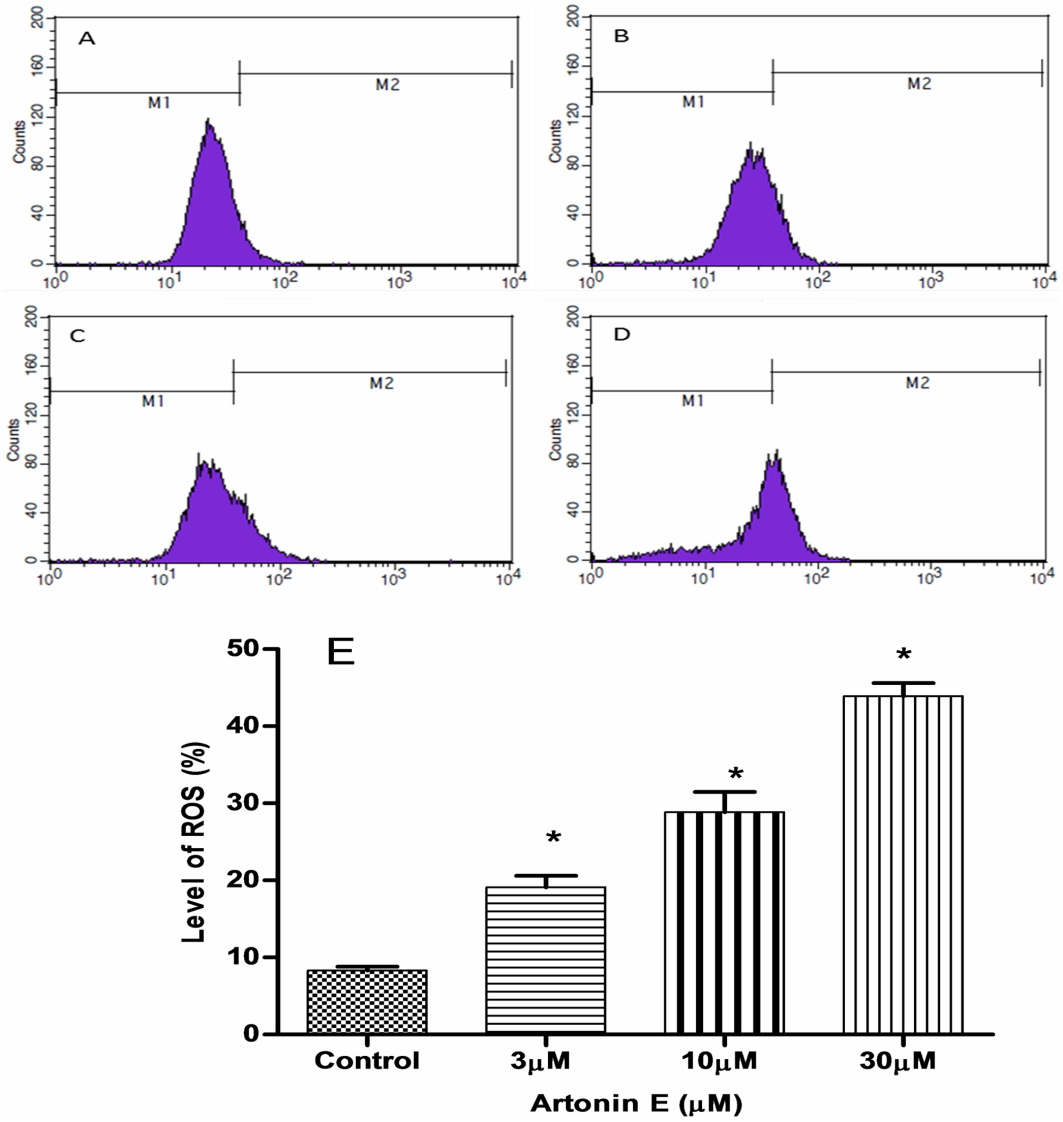
Total ROS production by MDA-MB 231 cells after treatment with Artonin E. (A) Untreated control and (B), (C) and (D) are treatment with 3, 10 and 30 μM Artonin E, respectively. (E) is the analysis of the flow cytometric data. Values are means ± standard deviation of three replicates. *Means differ significantly (p<0.05) from that of untreated control. M1 = non-ROS while M2 indicate the percentage of cells with an increase production of ROS.

A consistent increase in the total ROS was observed in a concentration dependent manner after treatment with Artonin E. The level of intracellular ROS increased significantly (p< 0.05) from 8.3% in the untreated MDA-MB 231 cells to 19.1, 28.8 and 43.9% in cells treated with 3, 10 and 30 μM Artonin E, respectively (Fig 8).

### Artonin E altered the expression of apoptosis and cell cycle-related genes in breast cancer cells

After 24 hour treatment of the MDA-MB 231 breast cancer cells with 10 μM of Artonin E, there was a differential expression of apoptosis and cell cycle- related genes as presented in Table 3.

**Table 3:** Apoptosis pathway- and cell cycle-related genes and protein expression in Artonin E treated MDA-MB 231 cells.

No.	Genes/Proteins	Relative expression [%Log(fold change)]	Regulation	Full name
**Genes**				
**1**	Caspase 7	73.3	Up	Cystein protease 7
**2**	Caspase 8	28.9	Up	Cystein protease 8
**3**	Caspase 9	92.2	Up	Cystein protease 9
**4**	Cyclin E	-55.6	Down	Cell cycle cyclin family member
**5**	P21	61.1	Up	Cyclin-dependent kinase inhibitor
**Proteins**				
**1.**	HSP 27	11.1	Up	Heat shock protein 27
**2.**	HSP 60	-13.5	Down	Heat shock protein 60
**3.**	HSP70	-55.8	Down	Heat shock protein 70
**4.**	Livin	-113.5	Down	Inhibitor of apoptosis protein
**5**	Bcl-2	-29.1	Down	B-cell lymphoma 2
**6.**	Bcl-x	-27.1	Down	Bcl-2-associated X protein
**7.**	Phospho S15	-17.6	Down	Phosphorylated p53 at serine residue 15
**8.**	Phopho S46	20.2	Up	Phosphorylated p53 at serine residue 46
**9.**	Phospho S392	26.7	Up	Phosphorylated p53 at serine residue 392
**10.**	SMAC/Diablo	23.8	Up	Second mitochondria-derived activator of caspases /direct IAP binding protein
**11.**	TRAIL	8.5	Up	TNF-related apoptosis-inducing ligand
**12**	HO-1/HMOX1/HSP32	5.6	Up	Heme oxygenase decyclig 1
**13**	cIAP-2	-18.8	Down	Cellular inhibitors of apoptosis
**14**	Pro-caspase 3	42.7	Up	Pro- Cysteine-aspartic acid protease 3
**15**	Cleaved caspase 3	-6.1	Down	Cleaved- Cysteine-aspartic acid protease 3

### Artonin E surpresses livin expression and upreguates p21 proteins dose dependently

**Fig 9:**
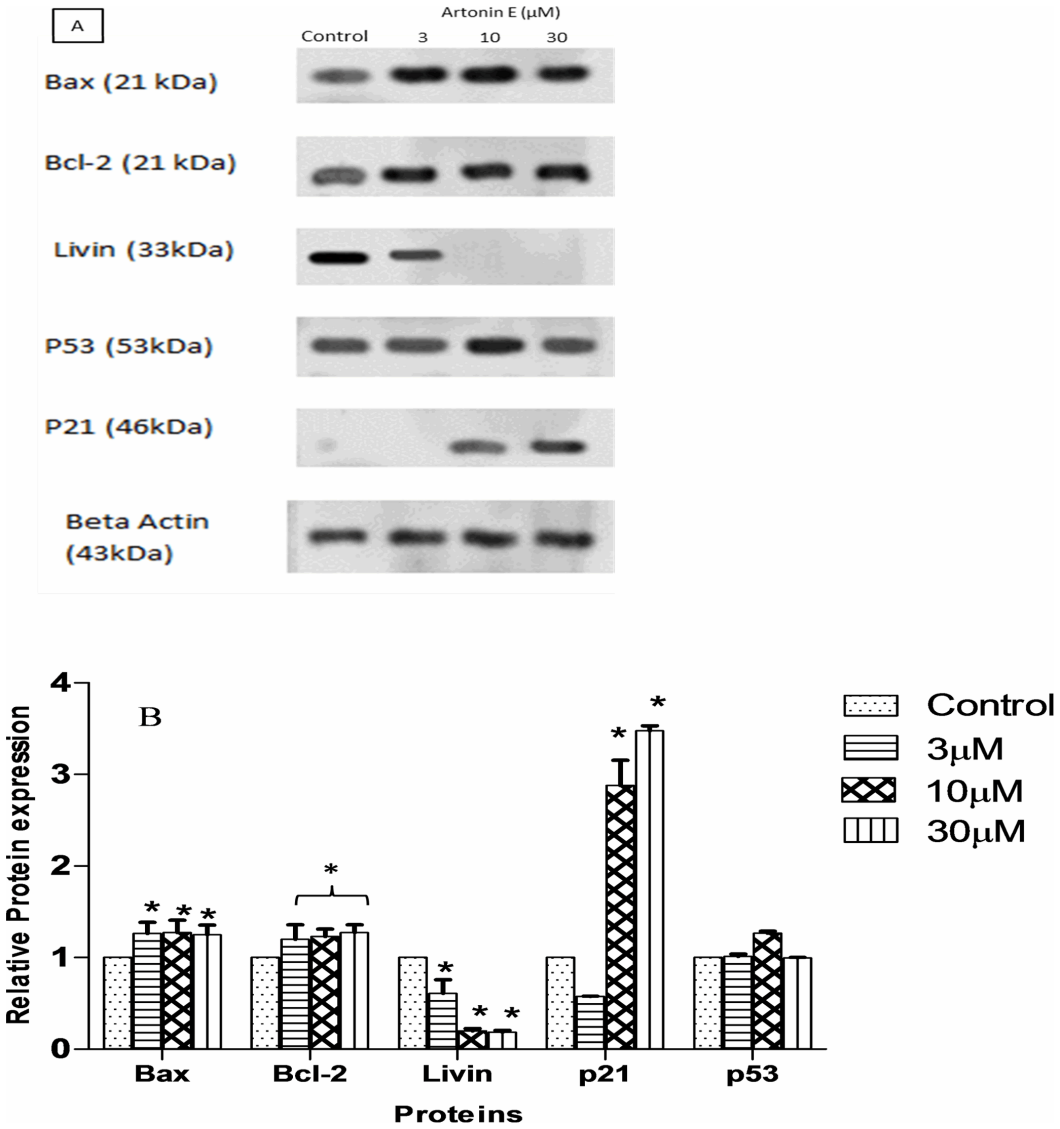
Western blot analysis of Artonin E (3, 10, and 30 μM) treated MDA-MB231 breast cancer cells after 24 hours. (A) and the levels of protein expression quantified from the western blotting analysis of Artonin E treated MDA-MB 231 cells using Bio-rad Image Lab software (B).

After treating the cancer cells with Artonin E, there was a dose dependent downregulation of livin, a new member of the inhibitors of apoptosis, which is responsible for breast cancer progression and invasiveness. A cyclin dependent kinase inhibitor, P21 was also confirmed to be upregulated after exposure of the triple negative breast cancer cells to Artonin E (Fig 9). Surprisingly, our findings revealed the suppression of Bcl-2 in the proteome array profiling but failed to validate it with the western blotting, which showed a rather slight induction of this anti-apoptotic protein after several replications. This discrepancy might be owed to a possible non-specific binding from the proteome array. The expression of p53 was rather unchanged in Artonin E treated and untreated MDA-MB 231 cells, which harbours a mutant copy of the protein.

## Discussion

Triple negative breast cancer, like other cancers, acquires certain capabilities which make it what it is, in addition to its unique heterogeneity and invasiveness. Prominent among these capabilities are its evasion of apoptosis and tissue invasion as well as metastasis[28]. These attributes coupled with the low rate of survival of patients bearing triple negative breast cancers, fueled the search for a promising candidate that can induce apoptosis and abrogate the intrinsic capabilities of the triple negative breast cancer. Previous literatures have reported the isolation of Artonin E from several species of Artocarpus as well as its cytotoxic effect on the growth of cancer cells, including the breast [29]. However, this cytotoxicity testing was only limited to the preliminary MTT assay and to the best of our knowledge, there is no report elucidating the possible molecular mechanism of action of Artonin E in MDA-MB 231 breast cancer cells. In this study, Artonin E which we had recently reported to be drug-like [23], was evaluated for its *in vitro* growth inhibition and molecular mechanism of cell death in MDA-MB 231 triple negative breast cancer cell line. Artonin E was found to significantly inhibit the proliferation of the breast cancer cells in a time and concentration dependent manner with a half maximal inhibitory concentrations of 14.13, 13.93 and 9.77 μM at 24, 48 and 72 hours, respectively. Artonin E showed a better selectivity (about 4.5 fold) for the MDA-MB 231 cancer cells than for the normal breast epithelial cells, MCF-10A in comparison to Tamoxifen, a standard agent (with a selectivity of 1.08). This attribute is in contrast to abounding standard treatments in the market which have been reported with negligible selectivity [30]. The less toxicity towards normal breast cells offers Artonin E a better therapeutic advantage over the standard agent, which in addition to negligible selectivity have also been reported with uprising resistance [31].

There are different modes of cell death, including apoptosis, necrosis and autophagy. From the results, the Artonin E-treated breast cancer cells displayed characteristic features of apoptosis. This was in accordance with a report by Carou *et al*. (2015)[32] and Gerl and Vaux (2005)[33], that apoptosis results in unique morphological changes like cell shrinkage, membrane alteration, DNA fragmentation and nuclear condensation. In fact, compounds that induce apoptosis are very essential in the management of cancer because evasion of apoptosis is implicated in cancer pathogenesis [28], [34] making its induction a strategy for cancer drug discovery[35].

The loss of membrane asymmetry during apoptosis leads to the externalization of phosphatidylserine. In this study, annexin V FITC and DNA binding flourochrome PI were utilized to further strengthen the assessment of the apoptotic mode of cell death and to examine the progression of apoptotic cells [12], [36], [37]. Artonin E was seen to significantly reduce the population of viable MDA-MB 231 breast cancer cells while increasing the population of cells undergoing apoptosis in a concentration dependent manner. These observations implicated apoptosis as the mode of cell death.

During apoptosis, chromosomal DNA is degraded by apoptotic endonucleases into fragments [38], which becomes visible when such DNA is run in a gel electrophoresis. Here, after treatment of the triple negative breast cancer cells, the cancer cell’s DNA was seen to have degraded as evidenced by the fragments visualized in the gel electrophoresis in comparison to the untreated control. This fragment induction by Artonin E, indicated an apoptotic cell death [27], which was deduced in previous assays above. In fact, the degradation of the cancer cells DNA discourages cell division, hence inhibiting the proliferation of the MDA-MB 231 cells.

Deregulation of cell cycle control has been evidently implicated in cancers [39–40]. This deregulation is often mediated by alterations in the activities of cyclin-dependent kinases that result in proliferation and chromosomal instability. Targeting the cell cycle and discovering inhibitors of the cyclin-dependent kinases are beneficial strategies in drug discovery [41]. In this study, the MDA-MB-231 which harbors mutant p53 was marginally arrested at the G2/M phase after treatment with Artonin E at 12 hours with a dose dependent increase in the sub G0/G1 phase. Artonin E-mediated G2/M phase arrest in MDA-MB 231 is suggested to be associated with the upregulation of p21 independent of p53 as was similarly pointed out [42]. In fact, Wafa and Pasumarthi (2015) [41], suggested that p21-mediated cell cycle inhibition can also occur via binding and inhibition of cyclins/Cdk. This cell cycle arrest prevented the MDA-MB-231 cells from undergoing cell division, purportedly because of the DNA fragmentation induced by Artonin E. The concentration- and time-dependent accumulation of the cancer cells at the subG0/G1 also suggested apoptosis [43]. To explore the mechanism by which Artonin E induced apoptosis, its effect on the initiators of caspase-dependent extrinsic and intrinsic pathways of apoptosis [44] was investigated. Caspase 8 is mostly responsible for the extrinsic pathway while caspase 9 is responsible for the intrinsic or mitochondrial pathways [45]. From this investigation, Artonin E activated both caspases 8 and 9 significantly in a concentration-dependent manner. These findings suggest that in MDA-MB-231, a triple negative breast cancer cell, the anticancer cell effect of Artonin E was via both the extrinsic and intrinsic or mitochondrial apoptotic pathways.

**Fig 10:**
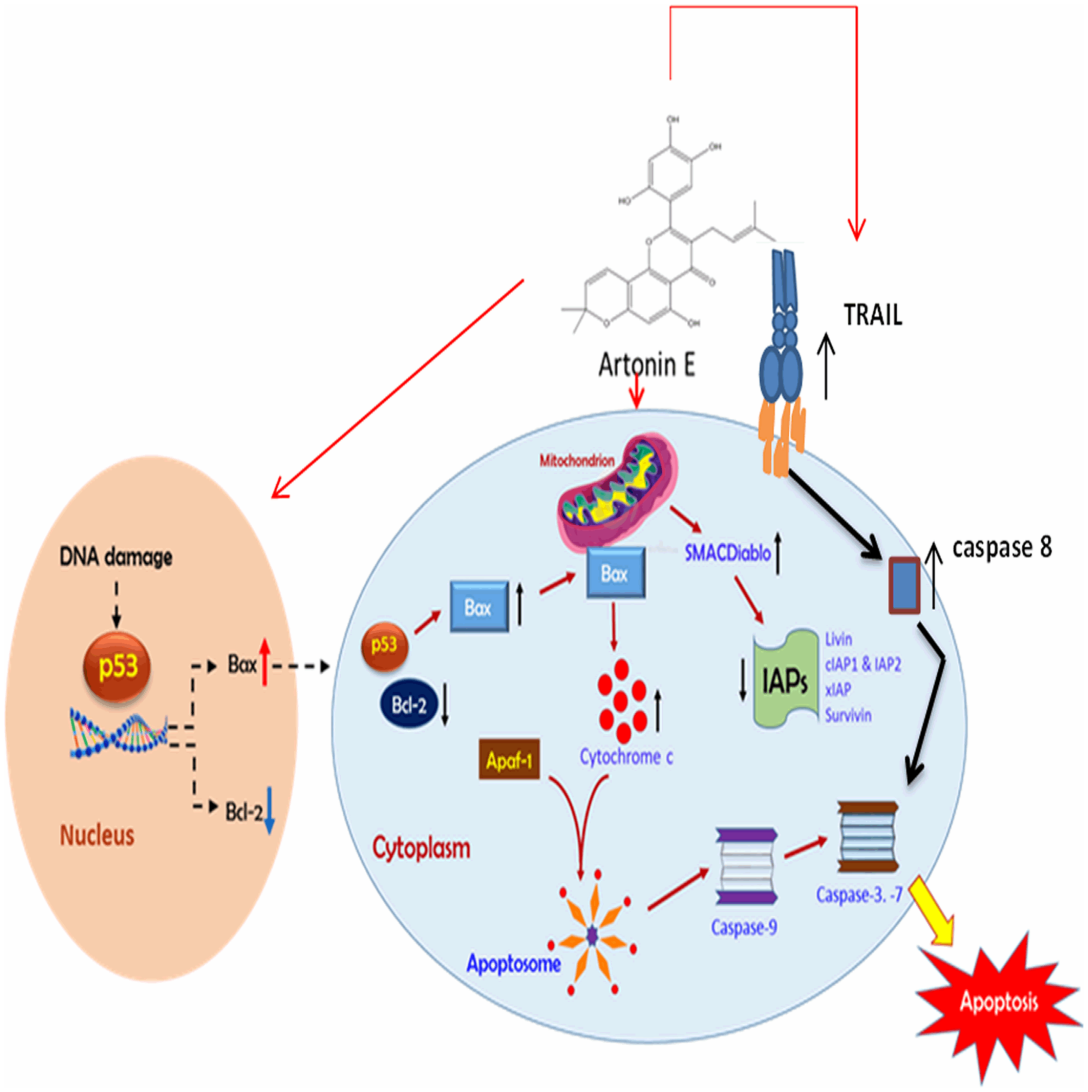
Mechanism of action of Artonin E in MDA-MB 231 breast cancer cells.

The mitochondrion is one of the main sources of ROS in cells and these chemicals regulate cell viability or mortality [12], [45–46]. Although oxygen metabolism is central to life, it has also been implicated in the development and spread of diseases like cancer [47–48]. However, beyond the cytotoxic concentration, ROS has been reported to trigger apoptosis [12], [49–50]. Unlike normal cells, cancer cells have restricted ability to nullify oxidative insults [51]. In this study, ROS production in Artonin E treated MDA-MB 231 cells was enhanced. The ROS level increased significantly from 8.3% in the untreated cells to 19.1, 28.8 and 43.9% after treatment with 3, 10 and 30 μM of Artonin E, respectively. This upregulation of ROS indicated that one of the mechanisms of apoptotic cell death induced by Artonin E treatment was via ROS production, an observation similar to that of Alexandre *et al*. (2006)[52]. In order to gain more insight into the molecular basis of the apoptotic signaling pathways in Artonin E-treated cancer cells, the gene and protein expressions were determined. From the results, the mRNA expression of caspases 7, 8 and 9 were upregulated. These are mediators of both the extrinsic and intrinsic pathways of apoptosis (Fig 10).

There was also an upregulation of the cyclin dependent kinase inhibitor, p21, which is suspected to have contributed to the earlier observed G2/M cell cycle arrest. Artonin E was seen to significantly inhibit the expression of anti-apoptotic proteins as well as inhibitors of apoptosis, when the human apoptosis proteome profiling was performed. From the results, Artonin E downregulated HSP 70, Bcl-x, livin and p53 phosphorylated at position 15 (S15). These proteins incidentally are overexpressed in aggressive breast cancers [52–53], indicating that the inhibition of expression of these proteins by Artonin E in MDA-MB 231 cells had relinquish these cancer cells of their inherent aggressiveness, enabling them to commit suicide. The observed downregulation of cIAP2, a G2/M phase cell cycle regulator [54–55], after treatment with Artonin E, had also been suspected as a contributing factor to the accumulation of MDA-MB 231 breast cancer cells in the G2/M phase [56]. Other proteins, like SMAC/Diablo, TRAIL and HO-1/HMOX1/HSP32 were upregulated in MDA-MB-231 treated with Artonin E. These proteins are either inhibitors of the action of apoptosis inhibitors or on their own they are inducers of apoptosis [57]. The significant upregulation of TRAIL, a tumor necrosis factor-related apotosis inducing ligand still showed the involvement of the extrinsic pathway in the apoptosis inducing effect of Artonin E. SMAC/DIABLO, a natural antagonist of the inhibitors of apoptosis [58] was found to be upregulated in Artonin E-treated MDA-MB-231 cells. The upregulation of SMAC/Diablo expression can cause cell cycle arrest and increase in caspase activities, which was part of the mechanism by which Artonin E inhibited breast cancer growth and induced apoptosis as observed earlier.

Western blot analysis revealed a significant (p<0.05) downregulation of livin, a novel member of apoptosis which has been reported as a marker of breast cancer progression and invasiveness [58–60]. This downregulation of livin is suspected to be responsible for the antiproliferative effect displayed by Artonin E in MDA-MB 231 cells. In an earlier experiment reported by Ou *et al*. (2014) [59], a gene knockdown of livin was shown to inhibit proliferation as well as invasiveness of cancer cells. Artonin E inhibition of livin had played a crucial role in the inhibition and apoptosis of the triple negative breast cancer cell line, MDA-MB 231.

## Conclusions

In conclusion, we have shown for the first time that Artonin E represses livin protein, upregulates p21 while activating caspase dependent ROS production in MDA-MB 231 triple negative breast cancer cell line. Artonin E, in its course to circumvent the relative immortality displayed by this cancer via apoptosis induction, affects the expression of apoptotic and cell cycle related genes and proteins which should be individually investigated in view of ascertaining if apoptosis will be averted in the knockdown of such genes or proteins. It is no doubt that Artonin E possesses the potentials that can be exploited to improve the poor prognosis of triple negative breast cancers.
